# Efficacy of goal-directed rehabilitation on functional recovery in patients with cerebral infarction

**DOI:** 10.1097/MD.0000000000047914

**Published:** 2026-03-27

**Authors:** Wenzhe Zhang, Meng Li, Bin Yang, Wenqian Zhang

**Affiliations:** a Department of Rehabilitation Medicine, Beijing Friendship Hospital, Capital Medical University, Beijing, China; b Department of Neurosurgery, North China University of Science and Technology Affiliated Hospital, Tangshan, Hebei, China; c Intensive Care Unit, the Department of Neurology, Affiliated Hospital of North China Polytechnic University, Tangshan, Hebei, China; d Department of Hepatobiliary and Pancreatic Surgery, North China University of Science and Technology Affiliated Hospital, Tangshan, Hebei, China.

**Keywords:** activities of daily living, Berg balance scale, cerebral infarction, Fugl–Meyer assessment, functional recovery, goal-directed rehabilitation, swallowing function

## Abstract

This retrospective cohort study evaluated the effectiveness of a structured goal-directed rehabilitation program compared with conventional rehabilitation in patients with cerebral infarction during inpatient recovery. A total of 185 patients admitted for rehabilitation between January 2023 and December 2024 were included. Apart from the implementation of a structured goal-directed rehabilitation framework in 2024, staffing structure, therapist training background, rehabilitation intensity, and equipment conditions remained stable across the study period. Functional outcomes were assessed at admission and discharge using standardized instruments including the Barthel index, Fugl–Meyer assessment, Berg balance scale, walking distance, walking speed, communication ability, and bedside swallowing evaluation. Both groups showed significant improvements across functional domains; however, the goal-directed rehabilitation group achieved larger gains in activities of daily living and motor function (Barthel index: +25.0 vs +18.0; fugl–meyer assessment: +16.0 vs +11.0; both *P* < .001). Improvements in balance, gait performance, communication, and swallowing were also more pronounced. Nevertheless, the findings reflect short-term inpatient rehabilitation outcomes from a single-center retrospective study and should be interpreted cautiously regarding long-term functional recovery and generalizability. These results suggest that integrating individualized, explicitly defined rehabilitation goals into multidisciplinary stroke rehabilitation may enhance short-term functional recovery, but prospective multicenter studies with longer follow-up are required to confirm these observations.

## 1. Introduction

Cerebral infarction is a major cause of long-term neurological disability worldwide and is frequently accompanied by persistent impairments in motor function, balance, gait, communication, and swallowing that restrict activities of daily living (ADL) and social participation. Contemporary stroke care increasingly recognizes rehabilitation as a core component of the continuum of management, with emphasis on organized, multidisciplinary program delivered within the framework of the International Classification of Functioning, Disability and Health. Recent narrative and systematic reviews have highlighted that structured stroke rehabilitation can improve functional outcomes, reduce disability, and support community reintegration, but they have also underscored substantial variability in how services are organized and delivered.^[1,2]^ A growing body of evidence indicates that early, intensive, and task-oriented rehabilitation is associated with better functional recovery after ischemic stroke. Meta-analyses and clinical studies have shown that appropriately timed early rehabilitation can improve neurological status, ADL performance, and limb motor function, although excessively aggressive very early mobilization may increase short-term risks and thus requires careful protocol design.^[3]^ Early rehabilitation nursing interventions have been reported to enhance neurological recovery, limb mobility, and mental health in stroke survivors, further supporting the importance of coordinated interdisciplinary care.^[4,5]^ In parallel, gait- and balance-focused interventions, including various forms of gait training, task-oriented exercises, and circuit-based program, have been shown to improve dynamic balance, walking speed, and functional mobility in subacute and chronic stroke populations.^[6,7]^ These data collectively support the principle that rehabilitation should be intensive, functionally oriented, and multidimensional.

Within this evolving landscape, goal-directed rehabilitation and person-centered goal setting have attracted increasing attention as potential means to enhance the effectiveness and relevance of stroke rehabilitation. Person-centered goal-setting frameworks aim to align rehabilitation content with patient-identified priorities in ADL, mobility, communication, and participation, while facilitating collaboration within multidisciplinary teams. Recent work has described and tested structured goal-setting packages designed to support comprehensive, person-centered goal setting in stroke rehabilitation, demonstrating feasibility and perceived benefit in both inpatient and community-based settings.^[8,9]^ Qualitative and survey-based studies have identified that engaging patients in goal setting can improve motivation, confidence, and satisfaction, but also revealed barriers related to time constraints, communication challenges, and limited training of rehabilitation professionals.^[10]^ Observational data from stroke units further show that patients frequently prioritize goals related to walking, ADL, and language, yet mismatches sometimes occur between documented goals and underlying impairments, indicating room for improvement in the systematic implementation of goal-directed approaches.^[11]^

Despite these advances, empirical data on comprehensive goal-directed rehabilitation as an organizing principle for routine inpatient program remain limited. Many existing studies focus on single modalities (e.g., upper-limb training, telerehabilitation) or particular phases of recovery, and few have evaluated goal-directed models that systematically integrate individualized goals into coordinated physiotherapy, occupational therapy, speech therapy, and nursing care across multiple functional domains.^[12]^ In this context, examining whether a structured, goal-directed rehabilitation program can enhance recovery of ADL, limb motor function, gait and balance, and communication and swallowing, compared with conventional therapist-directed rehabilitation of similar intensity in patients with cerebral infarction, is of practical importance for optimizing real-world stroke rehabilitation practice.

## 2. Materials and methods

### 2.1. Study design

This study was approved by the Ethics Committee of Affiliated Hospital of North China University of Science and Technology. This retrospective cohort study included patients diagnosed with cerebral infarction who received inpatient rehabilitation at our institution between January 2023 and December 2024. Patients admitted between January and December 2023 who underwent conventional rehabilitation formed the control group, whereas those admitted between January and December 2024 who received a structured goal-directed rehabilitation program constituted the observation group. The study was conducted in accordance with the principles of the Declaration of Helsinki and was approved by the institutional medical ethics committee. Written informed consent was obtained from all patients or their legally authorized representatives.

### 2.2. Inclusion and exclusion criteria

Patients were eligible for enrollment if they met all of the following conditions: age ≥18 years; diagnosis of cerebral infarction confirmed by cranial computed tomography or magnetic resonance imaging; first-ever ischemic stroke with residual motor and/or functional deficits requiring inpatient rehabilitation; admission to the rehabilitation unit within 3 months after stroke onset; hemodynamic stability after completion of acute-phase medical or interventional treatment; and availability of complete clinical and functional data at both admission and discharge required for the present analysis. Exclusion criteria were: intracerebral hemorrhage, subarachnoid hemorrhage, or transient ischemic attack without radiological evidence of infarction; severe preexisting disability before the index stroke, defined as a pre-stroke modified Rankin scale score ≥ 3; severe cognitive impairment, global aphasia, or major psychiatric disorders that precluded active participation in the rehabilitation program or reliable outcome assessment; concomitant progressive neurological diseases (such as Parkinson’s disease or multiple sclerosis), severe cardiac, hepatic, or renal dysfunction, or advanced malignancy with limited life expectancy; occurrence of another major neurological event, including recurrent stroke, during the rehabilitation period; and incomplete medical records or missing key baseline or follow-up outcome data.

To minimize potential confounding related to institutional resources, staffing patterns, therapist training background, rehabilitation intensity, and equipment availability remained essentially unchanged between 2023 and 2024. The same multidisciplinary rehabilitation team provided care during both periods, and no major organizational or technological changes were implemented apart from the introduction of the structured goal-directed rehabilitation framework. Rehabilitation session frequency, duration, and overall therapy intensity were maintained according to the same institutional standards across both groups.

### 2.3. Rehabilitation protocols in the conventional and goal-directed groups

All patients received standard medical management and secondary prevention of cerebral infarction according to contemporary guidelines, in addition to a structured rehabilitation program. In the conventional rehabilitation group, patients underwent a routine, therapist-directed program consisting mainly of physical therapy, occupational therapy, and nursing-led functional training. Physical therapy focused on limb range-of-motion exercises, muscle strengthening of the paretic side, balance and posture control, bed mobility, sitting and standing practice, transfer training, and basic gait training with appropriate assistive devices. Occupational therapy targeted upper-limb functional use, fine motor coordination, and training in basic ADL, including dressing, grooming, toileting, and feeding. When indicated, speech and swallowing therapy was provided for patients with dysarthria, aphasia, or dysphagia. Rehabilitation sessions were delivered once to twice daily, 5 to 6 d/wk, with each session lasting ~40 to 60 minutes, and the content and progression were mainly determined by the treating therapists’ clinical judgement and institutional routine, without formalized individual goal-setting procedures.

In the goal-directed rehabilitation group, patients received an enhanced, goal-oriented program on the basis of the same rehabilitation modalities. At admission, a comprehensive assessment of neurological deficits, motor function, ADL performance, balance, communication, cognition, and psychosocial status was performed using standardized scales (National Institutes of Health Stroke Scale [NIHSS], Fugl–Meyer assessment [FMA], Barthel index, Berg balance scale [BBS], and relevant cognitive and speech assessments). A multidisciplinary team comprising a rehabilitation physician, physical and occupational therapists, speech therapist, and specialist nurses conducted a structured meeting with the patient and main caregiver to identify priority functional problems and to formulate individualized rehabilitation goals using the SMART principles (specific, measurable, achievable, relevant, and time-bound). Long-term goals for the entire inpatient stay (e.g., independent indoor ambulation, independent toileting, or safe oral feeding) were decomposed into short-term weekly goals, and written goal sheets were documented in the medical record and communicated to all team members. Daily therapy sessions (once to twice per day, 5–6 d/wk, 40–60 min/session) were then explicitly planned and adjusted to address these predefined goals, emphasizing task-specific, repetitive training closely related to targeted real-life activities. Progress towards each goal was reviewed at least once weekly in team conferences, with quantitative re-assessment when necessary; goals and treatment plans were modified accordingly. Nursing staff reinforced goal-related practice during routine care (e.g., guided self-care training during washing, dressing, and toileting), and caregivers received structured education and bedside coaching to support continuation of goal-directed exercises outside formal therapy sessions.

### 2.4. Data collection

All data were obtained retrospectively from the institutional electronic medical record system and rehabilitation documentation using a predefined case report form. For each eligible patient, baseline demographic variables were collected at the time of admission to the rehabilitation unit, including age, sex, and body mass index, as well as lifestyle factors such as current smoking and alcohol use. Vascular risk factors and comorbidities were extracted from admission notes and discharge summaries and included the presence of hypertension, diabetes mellitus, dyslipidemia, atrial fibrillation, coronary artery disease, and a history of prior stroke. Stroke-related clinical information was collected from neurologist and radiology reports. This included the interval from stroke onset to rehabilitation admission (days), stroke etiology classified according to the Trial of Org 10172 in Acute Stroke Treatment criteria (large-artery atherosclerosis, cardioembolic, small-vessel occlusion, or other/undetermined causes), and lesion location (left hemisphere, right hemisphere, or brainstem/cerebellum) based on computed tomography or magnetic resonance imaging. Baseline neurological deficit was quantified using the NIHSS. Global motor impairment was assessed with the FMA motor score. ADL were evaluated using the Barthel index, and overall disability was graded using the modified Rankin scale (mRS). All baseline functional scales were administered by trained rehabilitation physicians or therapists within 24 hours of admission to the rehabilitation unit.

Functional outcomes were reassessed within 24 to 48 hours before discharge using the same instruments. For each patient, changes in Barthel index and FMA motor score (discharge minus admission) were calculated to quantify ADL and motor recovery over the course of inpatient rehabilitation. In addition, gait and balance performance were evaluated at both admission and discharge by physical therapists using the BBS, a standardized corridor walking test to record maximal walking distance over a fixed time interval, and a 10-m walking test to derive comfortable walking speed (m/s). The corresponding changes in BBS score, walking distance, and walking speed were derived for analysis. For patients documented to have communication or swallowing impairment at baseline, speech and swallowing function were systematically assessed by speech-language pathologists. Communicative ability was rated using a standardized ordinal communication scale routinely employed in the rehabilitation unit, with higher scores indicating better functional communication. Swallowing status was evaluated using a standardized bedside swallowing assessment, and the ability to resume safe oral feeding (yes/no) was recorded. For these patients, changes in communication score from admission to discharge and the proportion achieving a clinically meaningful improvement in communication, as well as the proportion regaining safe oral intake by discharge, were extracted for analysis. All collected data were anonymized and checked by 2 investigators independently, and only patients with complete baseline and discharge assessments for the relevant outcome measures were included in the final analyses.

### 2.5. Statistical analysis

All statistical analyses were performed using IBM SPSS Statistics, version 26.0 (IBM Corp., Armonk). Continuous variables were expressed as mean ± standard deviation when approximately normally distributed, and as median with interquartile range (IQR) for ordinal variables. Categorical variables were presented as counts and percentages. Baseline demographic and clinical characteristics were compared between the conventional rehabilitation group and the goal-directed rehabilitation group using independent-samples *t* tests for continuous variables and Pearson’s chi-square tests for categorical variables. The mRS, as an ordinal variable, was compared between groups using the Mann–Whitney *U* test. For functional outcomes, within-group changes from admission to discharge (e.g., Barthel index, FMA motor score, BBS, walking distance, walking speed, and communication scores) were analyzed using paired-samples t tests. Between-group differences in the magnitude of change (Δ = discharge − admission) were assessed using independent-samples *t* tests, and mean differences in change between groups were reported together with their 95% confidence intervals (CI). All statistical tests were 2-sided, and a *P*-value < .05 was considered to indicate statistical significance.

## 3. Results

### 3.1. Baseline demographic and clinical characteristics

Baseline demographic and clinical characteristics of the 185 included patients are summarized in Table 1. The 2 groups were well balanced at baseline. Mean age was comparable (66.2 ± 7.5 vs 66.4 ± 7.6 years, *P* = .857), and the proportion of male patients did not differ significantly (56.2% vs 61.5%, *P* = .562). Body mass index was similar between the conventional and goal-directed rehabilitation groups (24.0 ± 3.3 vs 24.4 ± 2.3 kg/m^2^, *P* = .344), as were the proportions of current smokers (37.1% vs 27.1%, *P* = .194) and current alcohol users (24.7% vs 28.1%, *P* = .720). The prevalence of major vascular risk factors and comorbidities was also comparable. There were no significant between-group differences in hypertension (68.5% vs 75.0%, *P* = .416), diabetes mellitus (30.3% vs 41.7%, *P* = .147), dyslipidemia (41.6% vs 37.5%, *P* = .678), atrial fibrillation (19.1% vs 19.8%, *P* = .906), coronary artery disease (28.1% vs 21.9%, *P* = .420), or prior stroke (28.1% vs 17.7%, *P* = .131; Table 1).

**Table 1:** Baseline demographic and clinical characteristics of the study population.

Variable	Conventional group (n = 89)	Goal-directed rehabilitation group (n = 96)	Test statistic*	*P*-value
Age (yr)	66.2 ± 7.5	66.4 ± 7.6	*t* = −0.18	.857
Male, n (%)	50 (56.2%)	59 (61.5%)	χ^2^ = 0.34	.562
BMI, kg/m^2^	24.0 ± 3.3	24.4 ± 2.3	*t* = −0.95	.344
Current smoking, n (%)	33 (37.1%)	26 (27.1%)	χ^2^ = 1.69	.194
Current alcohol use, n (%)	22 (24.7%)	27 (28.1%)	χ^2^ = 0.13	.720
Hypertension, n (%)	61 (68.5%)	72 (75.0%)	χ^2^ = 0.66	.416
Diabetes mellitus, n (%)	27 (30.3%)	40 (41.7%)	χ^2^ = 2.10	.147
Dyslipidaemia, n (%)	37 (41.6%)	36 (37.5%)	χ^2^ = 0.17	.678
Atrial fibrillation, n (%)	17 (19.1%)	19 (19.8%)	χ^2^ = 0.01	.906
Coronary artery disease, n (%)	25 (28.1%)	21 (21.9%)	χ^2^ = 0.65	.420
Prior stroke, n (%)	25 (28.1%)	17 (17.7%)	χ^2^ = 2.28	.131
TOAST subtype **−** LAA, n (%)	39 (43.8%)	33 (34.4%)		
TOAST subtype **−** CE, n (%)	15 (16.9%)	22 (22.9%)	χ^2^ = 3.85	.278
TOAST subtype **−** SVO, n (%)	32 (36.0%)	33 (34.4%)		
TOAST subtype **−** Other, n (%)	3 (3.4%)	8 (8.3%)		
Lesion location **−** left hemisphere, n (%)	50 (56.2%)	47 (49.0%)		
Lesion location **−** right hemisphere, n (%)	32 (36.0%)	41 (42.7%)	χ^2^ = 1.01	.605
Lesion location **−** brainstem/cerebellum, n (%)	7 (7.9%)	8 (8.3%)		
Onset to rehabilitation admission (d)	18.1 ± 7.5	16.2 ± 6.9	*t* = 1.79	.075
NIHSS score at admission	8.1 ± 3.1	7.8 ± 2.9	*t* = 0.68	.498
FMA motor score at admission	46.2 ± 14.4	49.6 ± 13.7	*t* = −1.64	.102
Barthel index at admission	50.8 ± 20.1	54.0 ± 20.2	*t* = −1.08	.282
mRS at admission, median (IQR)	3 (3–4)	3 (2–4)	Mann–Whitney *U*	.182

ADL = activities of daily living, BMI = body mass index, CE = cardioembolism, FMA = Fugl–Meyer assessment, IQR = interquartile range, LAA = large-artery atherosclerosis, mRS = modified Rankin scale, NIHSS = National Institutes of Health Stroke Scale, SVO = small-vessel occlusion, TOAST = Trial of Org 10172 in Acute Stroke Treatment.

Stroke-related characteristics at admission were similarly distributed. The time from stroke onset to rehabilitation admission showed a non-significant trend toward a slightly longer interval in the conventional group (18.1 ± 7.5 vs 16.2 ± 6.9 days, *P* = .075). Baseline neurological deficit and functional status were comparable, with similar NIHSS scores (8.1 ± 3.1 vs 7.8 ± 2.9 points, *P* = .498), Fugl–Meyer motor scores (46.2 ± 14.4 vs 49.6 ± 13.7 points, *P* = .102), and Barthel index scores (50.8 ± 20.1 vs 54.0 ± 20.2 points, *P* = .282). Median mRS at admission was 3 (IQR 3–4) in the conventional group and 3 (IQR 2–4) in the goal-directed rehabilitation group (*P* = .182). The distribution of Trial of Org 10172 in Acute Stroke Treatment stroke subtypes (large-artery atherosclerosis, cardioembolic, small-vessel occlusion, and other causes; overall χ^2^ = 3.85, *P* = .278) and lesion location (left hemisphere, right hemisphere, brainstem/cerebellum; χ^2^ = 1.01, *P* = .605) did not differ significantly between groups, indicating that the baseline vascular risk profile and stroke severity were well balanced.

### 3.2. Functional improvement from admission to discharge

From admission to discharge, both groups exhibited significant gains in ADL and motor function, with a greater magnitude of improvement in the goal-directed rehabilitation group. In the conventional rehabilitation group, the mean change in Barthel index (discharge minus admission) was 18.0 ± 10.0 points (*t* = 16.98, *P* < .001), and the mean change in FMA motor score was 11.0 ± 7.0 points (*t* = 14.82, *P* < .001). In the goal-directed rehabilitation group, the corresponding mean changes were 25.0 ± 11.0 points for the Barthel index (*t* = 22.27, *P* < .001) and 16.0 ± 8.0 points for the FMA motor score (*t* = 19.60, *P* < .001). Between-group comparisons showed that the mean difference in Barthel index change was 7.0 points (25.0 ± 11.0 vs 18.0 ± 10.0), with an estimated mean difference of 7.0 points (95% CI: 4.0–10.0; t = 4.53; *P* < .001). For the FMA motor score, the mean difference in change between the goal-directed and conventional rehabilitation groups was 5.0 points (16.0 ± 8.0 vs 11.0 ± 7.0), corresponding to a mean difference of 5.0 points (95% CI: 2.8–7.2; *t* = 4.53; *P* < .001). These results indicate that, although both rehabilitation strategies improved functional outcomes, goal-directed rehabilitation produced larger improvements in ADL and motor function (Table 2).

**Table 2:** Changes in functional outcomes from admission to discharge.

Outcome	Group	Change in score (Δ), mean ± SD	Within-group *t*	Within-group *P*-value	Between-group mean difference in change (95% CI)	Between-group *t*	Between-group *P*-value
Barthel index	Conventional	18.0 ± 10.0	*t* = 16.98	<.001			
	Goal-directed	25.0 ± 11.0	*t* = 22.27	<.001	7.0 (4.0–10.0)	*t* = 4.53	<.001
FMA motor score	Conventional	11.0 ± 7.0	*t* = 14.82	<.001			
	Goal-directed	16.0 ± 8.0	*t* = 19.60	<.001	5.0 (2.8–7.2)	*t* = 4.53	<.001

CI = confidence interval, FMA = Fugl–Meyer assessment.

### 3.3. Walking, balance, and motor performance

Both groups demonstrated significant improvements in balance and walking performance; however, improvements were more pronounced in the goal-directed rehabilitation group. The mean increase in BBS score was 9.0 ± 6.0 points in the conventional group (*t* = 14.15, *P* < .001) and 13.0 ± 7.0 points in the goal-directed group (*t* = 18.20, *P* < .001). The between-group difference in BBS change was 4.0 points, with a mean difference of 4.0 points (95% CI: 2.1–5.9; *t* = 4.18; *P* < .001). Walking distance from admission to discharge increased by 85.0 ± 60.0 m in the conventional group (*t* = 13.36, *P* < .001) and by 130.0 ± 70.0 m in the goal-directed group (*t* = 18.20, *P* < .001). The mean between-group difference in walking distance change was 45.0 m (95% CI: 26.2–63.8; *t* = 4.70; *P* < .001). Walking speed similarly improved in both groups, with a mean increase of 0.24 ± 0.18 m/s in the conventional group (*t* = 12.58, *P* < .001) and 0.36 ± 0.20 m/s in the goal-directed group (*t* = 17.64, *P* < .001). The mean between-group difference in walking speed change was 0.12 m/s (95% CI: 0.06–0.18; *t* = 4.29; *P* < .001), favoring goal-directed rehabilitation (Table 3).

**Table 3:** Balance and gait outcomes from admission to discharge.

Outcome	Group	Change in score (Δ), mean ± SD	Within-group *t*	Within-group *P*-value	Between-group mean difference in change (95% CI)	Between-group *t*	Between-group *P*-value
BBS score	Conventional	9.0 ± 6.0	*t* = 14.15	<.001			
	Goal-directed	13.0 ± 7.0	*t* = 18.20	<.001	4.0 (2.1–5.9)	*t* = 4.18	<.001
Walking distance, m	Conventional	85.0 ± 60.0	*t* = 13.36	<.001			
	Goal-directed	130.0 ± 70.0	*t* = 18.20	<.001	45.0 (26.2–63.8)	*t* = 4.70	<.001
Walking speed, m/s	Conventional	0.24 ± 0.18	*t* = 12.58	<.001			
	Goal-directed	0.36 ± 0.20	*t* = 17.64	<.001	0.12 (0.06–0.18)	*t* = 4.29	<.001

BBS = Berg Balance scale, CI = confidence interval.

### 3.4. Speech and swallowing function

In patients with communication and swallowing impairment at baseline, both groups showed improvement in communicative ability, with larger gains in the goal-directed rehabilitation group. The mean change in communication score (higher scores indicating better communication) was 2.5 ± 1.8 points in the conventional group (*t* = 13.10, *P* < .001) and 3.6 ± 2.0 points in the goal-directed group (*t* = 17.64, *P* < .001). The mean between-group difference in change was 1.1 points (95% CI: 0.55–1.65; *t* = 3.94; *P* < .001). A clinically meaningful improvement in communication, defined as an increase of ≥2 points, was observed in 46.1% (41/89) of patients in the conventional group and 65.6% (63/96) of patients in the goal-directed group (χ^2^(1) = 7.18, *P* = .007). Swallowing function also improved in both cohorts, with a higher proportion of patients recovering safe oral intake by discharge in the goal-directed rehabilitation group. The proportion of patients able to resume safe oral feeding was 68.5% (61/89) in the conventional group and 82.3% (79/96) in the goal-directed group (χ^2^ = 4.75, *P* = .029), indicating a significantly higher rate of swallowing recovery with goal-directed rehabilitation (Table 4).

**Table 4:** Communication and swallowing outcomes.

Outcome	Group	Change in score (Δ), mean ± SD/ n (%)	Test statistic	*P*-value
Communication score	Conventional	2.5 ± 1.8	*t* = 13.10 (88)	<.001
	Goal-directed	3.6 ± 2.0	*t* = 17.64 (95)	<.001
Between-group difference in communication score change	**−**	Mean difference 1.1 (95% CI 0.55–1.65)	*t* = 3.94 (≈181)	<.001
Clinically meaningful communication improvement (Δ ≥ 2), n (%)	Conventional	41 (46.1%)		
	Goal-directed	63 (65.6%)	χ^2^ = 7.18	.007
Safe oral intake at discharge, n (%)	Conventional)	61 (68.5%)		
	Goal-directed	79 (82.3%)	χ^2^ = 4.75	.029

SD = standard deviation.

## 4. Discussion

This retrospective cohort study evaluated the effect of a goal-directed rehabilitation program on multidimensional functional recovery in patients with cerebral infarction. Both conventional and goal-directed rehabilitation were associated with significant improvements in ADL, limb motor function, gait, balance, communication, and swallowing. However, patients receiving goal-directed rehabilitation achieved consistently greater gains across all functional domains, including larger increases in Barthel index and FMA motor scores, greater improvements in BBS scores, walking distance and walking speed, and higher rates of clinically meaningful communication improvement and recovery of safe oral intake. These findings suggest that structuring rehabilitation around individualized, explicitly defined functional goals may enhance the effectiveness of multidisciplinary stroke rehabilitation in the subacute phase.

The observed differences in ADL and motor recovery are clinically relevant. A 7-point higher improvement in Barthel index and a 5-point greater gain in FMA motor score in the goal-directed group indicate not only statistically significant but also functionally meaningful benefits, particularly in the context of inpatient rehabilitation where relatively short time frames limit the achievable magnitude of change. The superiority of goal-directed rehabilitation in BBS, walking distance, and walking speed suggests that framing therapy tasks around concrete mobility and participation goals may improve the integration of strength, balance, and motor control into functional ambulation, rather than producing isolated gains in impairment-level measures alone. The improvements in communication and swallowing outcomes are noteworthy. Patients in the goal-directed group demonstrated larger gains in communication scores and a higher proportion achieving a clinically meaningful ≥2-point improvement, indicating that explicit communication-related goals, combined with coordinated input from speech therapists and nursing staff, may enhance the translation of impairment-level gains into functional communicative ability. Similarly, higher rates of recovery of safe oral intake in the goal-directed group suggest that goal-focused swallowing management, including coordinated adjustments of diet texture, posture, and compensatory strategies during routine nursing care, can increase the effectiveness of dysphagia rehabilitation.

Several mechanisms may underlie the superior outcomes observed with goal-directed rehabilitation. First, the systematic use of structured, patient-centered goals based on the International Classification of Functioning, Disability and Health framework aligns rehabilitation content directly with meaningful activities and participation domains, potentially enhancing the relevance and intensity of training. Second, explicit goal-setting clarifies priorities for the multidisciplinary team, reducing redundancy and ensuring that therapy modalities are coordinated toward shared targets rather than operating in parallel silos. Third, involving patients and caregivers in the goal-setting process may improve motivation, engagement, and adherence to both supervised and unsupervised practice, which are recognized determinants of motor relearning and neuroplasticity. Finally, the requirement for regular review and adjustment of goals may promote a more responsive, data-driven adaptation of therapy intensity and content as patients progress.

Current stroke rehabilitation guidelines emphasize individualized, person-centered goal setting as a core component of high-quality rehabilitation, although the strength of evidence for specific goal-directed models has been considered moderate. The 2023 NICE guideline on stroke rehabilitation explicitly recommends collaborative goal-setting and regular goal review as key organizational principles to enhance patient engagement and optimize functional outcomes. Our results support and extend these recommendations by providing quantitative evidence that an explicitly structured goal-directed protocol is associated with greater gains in ADL and motor function than conventional therapist-directed rehabilitation of similar intensity. Several recent studies and reviews have highlighted the potential benefits of person-centered goal setting in neurological rehabilitation. A systematic review of patient-centered goal setting in stroke rehabilitation reported that shared goal-setting interventions can improve patient participation, satisfaction, and, in some studies, functional outcomes, although heterogeneity in interventions limited firm conclusions.^[13]^ A more recent review of person-centered goal-setting interventions across adult rehabilitation populations found that structured goal-setting frameworks incorporating explicit collaboration, documentation, and regular review were associated with improved goal attainment and functional performance.^[14]^ Emerging work on interdisciplinary goal-setting processes in specialist rehabilitation hospitals further suggests that formalized goal-setting can improve team coordination and clarify treatment priorities.^[9]^ Our findings are consistent with this body of evidence and add quantitative support specifically in the context of inpatient stroke rehabilitation.

With respect to mobility-related outcomes, our observation that goal-directed rehabilitation produced larger improvements in gait and balance aligns with the broader literature on task-oriented and high-dose training after stroke. Recent comprehensive reviews have concluded that task-specific, activity-focused physical therapy interventions are effective in improving ADL and functional mobility in stroke survivors, particularly when delivered at sufficient intensity.^[15,16]^ In parallel, recent trials and systematic reviews of dual-task and vestibular-focused training have demonstrated that targeted interventions can significantly enhance gait speed, balance, and mobility in subacute and chronic stroke populations.^[17]^ Although the present study did not isolate specific task-oriented or dual-task components, the greater improvements in BBS, walking distance, and walking speed in the goal-directed group are consistent with the hypothesis that framing training around functionally meaningful mobility goals leads to more effective practice of task-specific gait and balance activities.

Motivational and behavioral mechanisms may also contribute to the observed benefits. Recent neurorehabilitation literature has emphasized the central role of self-efficacy and motivation in stroke recovery, with systematic reviews demonstrating robust associations between self-efficacy and outcomes such as ADL, mobility, and quality of life.^[18]^ Experimental work and qualitative studies on goal-setting in stroke rehabilitation have shown that collaboratively defined goals can enhance patient motivation, perceived autonomy, and engagement in therapy.^[19,20]^ These constructs are particularly relevant to goal-directed interventions, which explicitly link daily therapy tasks to personally meaningful functional targets and encourage active patient participation in monitoring progress. The larger functional gains observed in our goal-directed group are therefore consistent with these mechanistic insights and suggest that structured goal-setting may operate both through improved technical targeting of therapy and through enhanced motivational and behavioral pathways. Our findings also resonate with recent narrative reviews arguing for a shift towards “precision rehabilitation” after stroke, in which interventions are tailored to individual profiles of impairment, activity limitation, and personal goals, rather than relying on uniform protocols.^[1]^ In this context, the present study provides empirical support for a relatively pragmatic goal-directed framework that could be integrated into existing multidisciplinary rehabilitation services without requiring advanced technology or complex resource inputs.

Several limitations should be acknowledged. First, the retrospective, non-randomized design precludes definitive causal inference. Allocation to conventional versus goal-directed rehabilitation was based on calendar time, which introduces potential confounding by secular changes in staffing, processes of care, or institutional experience, even though baseline demographic and clinical characteristics were balanced between groups. Second, the study was conducted in a single center, which may limit generalizability to settings with different organizational structures, resource levels, or patient populations. Third, follow-up was limited to the inpatient rehabilitation period; no data were available on longer-term outcomes such as community participation, recurrent vascular events, or sustained functional independence after discharge. Fourth, although validated scales were used for most outcomes, the communication measure was a unit-specific ordinal scale rather than a widely standardized instrument, and the swallowing assessment was based on bedside evaluation rather than instrumental studies, which may introduce measurement variability. Finally, the study did not formally quantify rehabilitation dose (e.g., exact minutes of task-specific training) or patient-reported outcomes such as satisfaction or self-efficacy, which could help clarify the mechanisms by which goal-directed rehabilitation exerts its effects. In addition, although widely used functional scales such as the Barthel index, FMA, and BBS have established validity, their repeatability may vary across evaluators and institutions, particularly in routine clinical settings without centralized training or calibration. Differences in rehabilitation infrastructure, staff expertise, patient case mix, and health system organization may also influence the feasibility and effectiveness of goal-directed rehabilitation models. Therefore, caution is warranted when extrapolating these findings to other centers, and multicenter prospective studies with standardized assessment protocols are needed to confirm reproducibility and external validity.

## 5. Conclusion

In this retrospective study of patients with cerebral infarction, both rehabilitation models improved ADL, motor function, gait, balance, communication, and swallowing during the inpatient rehabilitation period. Goal-directed rehabilitation was associated with consistently greater short-term functional gains across multiple domains. However, these findings primarily reflect short-term inpatient rehabilitation effects from a single-center retrospective cohort and should not be generalized to long-term functional outcomes or broader clinical settings without further prospective, multicenter studies with extended follow-up.

## Author contributions

**Conceptualization:** Wenzhe Zhang, Meng Li, Wenqian Zhang.

**Data curation:** Wenzhe Zhang, Meng Li, Bin Yang, Wenqian Zhang.

**Formal analysis:** Wenzhe Zhang, Meng Li, Bin Yang, Wenqian Zhang.

**Funding acquisition:** Wenzhe Zhang, Bin Yang, Wenqian Zhang.

**Investigation:** Wenqian Zhang.

**Writing − original draft:** Wenzhe Zhang, Wenqian Zhang.

**Writing − review & editing:** Wenzhe Zhang, Wenqian Zhang.
